# The Impact of the Menstrual Cycle on Perioperative Bleeding in Vitreoretinal Surgery

**DOI:** 10.1155/2017/9549284

**Published:** 2017-03-16

**Authors:** Zhong Lin, Nived Moonasar, Rong Han Wu, Robin R. Seemongal-Dass

**Affiliations:** ^1^The Eye Hospital, School of Ophthalmology and Optometry, Wenzhou Medical University, Wenzhou, Zhejiang, China; ^2^Ophthalmology Unit, Department of Surgery, Faculty of Medical Sciences, University of the West Indies, St. Augustine, Trinidad and Tobago

## Abstract

*Purpose.* To investigate the effect of menstrual cycle on perioperative bleeding of primary vitreoretinal surgery. *Methods.* Data on female patients who had vitrectomy surgery was retrospectively collected. Exclusion criteria were history of trauma, vitreous hemorrhage, previous vitreoretinal surgery, diabetic retinopathy, endophthalmitis, acute retinal necrosis, single vitreous opacity, and use of antiplatelet agents. Perioperative bleeding was defined as hemorrhage in the iris, vitreous, choroidal, retina, or subretina during surgery or up to one day postoperatively. 69 patients had surgery during the perimenstrual phase (group M, days 1–7 and days 21–28) and 86 during periovulatory phase (group O, days 8–20) were enrolled. *Results.* The proportion of operative bleeding in group M (14.5%) and group O (10.5%) was not found to be significantly different (*p* = 0.45). No postoperative bleeding was recorded in both groups. The univariate odds ratio (OR) and 95% confidence interval (CI) of perimenstrual phase for operative bleeding were 0.69 (0.26–1.81). After adjusting for patients' age, vitreoretinal diseases, and surgeons, the multivariate OR and 95% CI were 0.71 (0.27–1.86). *Conclusion.* This study suggests that the timing of the menstrual period does not affect perioperative bleeding for primary vitreoretinal surgery. Menstruation appears not to be a contraindication for vitreoretinal surgery.

## 1. Introduction

Among some surgeons, there is a belief that menstruation is a contraindication to elective surgery. This has been attributed to, among other things, functional impairment of the coagulation system during menstruation [1]. Recent studies have demonstrated that hormonal changes can induce perioperative bleeding, especially in the hormonally dependent organs of female patients. Studies on rhinoplasty and breast reduction surgery have shown significantly greater blood loss during the periovulatory or perimenstrual phase [2, 3]. However, with respect to hormonally independent organs, one study showed no differences of intra- and postoperative blood loss of abdominoplasty, during perimenstrual (0–7, 21–28 days), periovulatory (8–20 days), and postmenopausal periods [4]. To our knowledge, there is no study concerning the perioperative bleeding of vitreoretinal surgery during the menstrual cycle. Hence, this study aims to investigate the effect of the menstrual cycle on the perioperative bleeding during primary vitreoretinal surgery.

## 2. Methods

Data on female patients who had vitrectomy from January 2004 to December 2013 at the Eye Hospital of Wenzhou Medical University were retrospectively collected. All the information, including general, ocular, medical, and surgical history and preoperative and postoperative ophthalmic examinations, was obtained from electronic medical records. The inclusion criteria were females who had vitrectomy and had normal menstrual cycles. The exclusion criteria were (1) previous ovarian, uterine, or vaginal surgery that may affect menstruation; (2) history of ocular penetrating trauma; (3) previous vitreoretinal surgery in the same eye; (4) vitreous hemorrhage; (5) any vitreoretinal vascular diseases such as diabetic retinopathy, choroidal neovascularization, and polypoidal choroidal vasculopathy that may have influenced the perioperative bleeding; (6) single vitreous opacity; (7) endophthalmitis or acute retina necrosis; (8) incomplete information on medical information; and (9) use of aspirin and/or other antiplatelet agents. Previous childbirth was not an exclusion criterion.

All patients had vitrectomy by one of 5 surgeons. Pars plana vitrectomy (PPV) was performed using either a 23-gauge or a 20-gauge vitrectomy system. Operative and postoperative surgical bleeding was defined as hemorrhage in the iris, vitreous, choroid, retina, or subretina during surgery or postoperatively up to one day.

**Figure 1:**
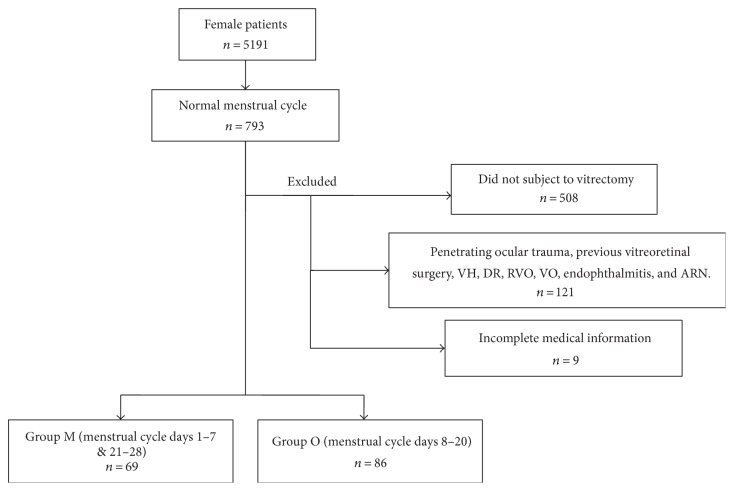
The flow chart of enrolled patients. VH: vitreous hemorrhage; DR: diabetic retinopathy; RVO: retina vein occlusion; VO: vitreous opacity; and ARN: acute retina necrosis. Group M: perimenstrual phase; group O: preovulatory phase.

A total of 5191 female patient records were examined. 793 women with a normal menstrual cycle had ocular surgery. Of these, 508 did not have vitrectomy; 121 had penetrating ocular trauma and/or previous vitreoretinal surgery or vitreous hemorrhage, diabetic retinopathy, and/or other vitreoretinal vascular disease (such as retina vein occlusion) or performed vitrectomy for single vitreous opacity, endophthalmitis, or acute retina necrosis; and 9 with incomplete medical information were excluded. This resulted in 155 patients being enrolled. Among these patients, 69 during the perimenstrual phase (days 1–7 and days 21–28) and 86 during the periovulatory phase (days 8–20) were categorized into group M and group O, respectively (Figure 1).

The normally distributed parameters were presented as the mean ± standard deviation, while the and non-normally distributed parameters were presented as median and quartile ranges. Both univariate and multivariate logistic analyses were conducted to access the relationship between menstruation day and operative bleeding. The odds ratio (OR) and 95% confidence interval (CI) were presented. Statistical analysis was performed with Statistical Analysis System for Windows version 9.1.3 (SAS Inc., Cary, NC).

## 3. Results

The characteristics of these two groups are presented in Table 1. The mean age was 42.0 ± 11.4 and 41.0 ± 9.7 years (*p* = 0.55) in group M and group O, respectively. Vitreoretinal disease types were similar in both groups (*p* = 0.83), while rhegmatogenous retinal detachment was the main type (>65%). Surgeons' distribution was also not significantly different. Postoperative bleeding was not recorded in either group.

**Figure 2:**
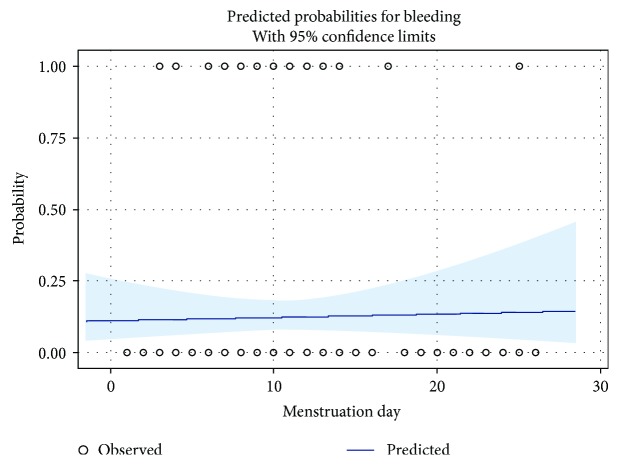
Predicted probability for perioperative bleeding as a function of menstruation day.

There were 10 group M patients and 9 group O patients with operative bleeding. All were recorded as punctate bleeding on the retina. The punctate bleeding was handled with electrocoaguation in both groups. The proportion of the operative bleeding was not significantly different between group M and group O (14.5% and 10.5%, *p* = 0.45). When taking the operative bleeding as the dependant variable, univariate logistic analysis showed neither day of menstruation (OR, 95% CI 1.01, 0.93–1.10) nor menstruation group (OR, 95% CI 0.69, 0.26–1.81) was significant for operative bleeding. When taking patients' age, vitreoretinal disease, and surgeon into account, similar results were found in the multivariate logistic analysis, that is, neither day of menstruation (OR, 95% CI 1.00, 0.92–1.09) nor menstruation group (OR, 95% CI 0.71, 0.27–1.86) was significant for operative bleeding (Table 2). The predicted probabilities for perioperative bleeding as a function of each menstruation day is also presented (Figure 2).

## 4. Discussion

Surgical bleeding is one of the main challenges faced by surgeons that directly affects morbidity and mortality as well as surgeon and patient comfort. Kenig et al. summarized previous studies and found that the menstrual cycle may influence perioperative pain [5], blood loss [2, 3], or recurrence/mortality [6] in hormonally dependent organs, such as the vagina, breasts, oronasal mucosa, and Eustachian tube. However, there was no significant effect on the hormonally independent organs, such as the abdominal region.

To our knowledge, there is no evidence showing that the tissue of the iris, choroid, or retina is under the direct influence of hormonal changes during the menstrual cycle. Despite this, some surgeons may hold on to a traditional belief that menstruation is a contraindication to surgery of any type including vitrectomy. As far as we are aware, this belief has not been backed up by scientific evidence.

In this study, vitreoretinal diseases that would apparently increase the chance of perioperative bleeding, such as vitreous hemorrhage and any vitreoretinal vascular diseases, were excluded. We also excluded vitrectomy for single vitreous opacity since it may decrease the perioperative bleeding proportion. In this study, we found though the proportion of operative bleeding was slightly higher in the perimenstrual phase than in the periovulatory phase (14.5% versus 10.5%), it was not statistically or clinically significant. Both univariate and multivariate logistic analyses showed no effect of the menstrual cycle on operative bleeding. This suggests that the menstrual cycle has no effect on operative bleeding on primary vitreoretinal surgery. We also found no significant effect of patients' age or vitreoretinal disease type. With respect to hypertension, due to a very small sample size (less than ten hypertensive patients), this risk factor could not be analyzed. As there was no significant effect of the menstrual cycle on operative bleeding, we recommend that menstruation should not be a contraindication to vitrectomy.

Limitations of this study included data inaccuracy due to its retrospective analysis and recall bias of patients with respect to the menstrual data. However, considering the clinical feasibility of the interpretation, we believe this would not have a great influence on the conclusion. However, further prospective studies may be warranted.

## Conflicts of Interest

The authors have no proprietary or commercial interest in any materials discussed in this article.

## Figures and Tables

**Table 1:** Characteristics for patients during perimenstrual phase (group M) and periovulatory phase (group O).

	Group M *N* = 69	Group O *N* = 86	*p*
Age (years, mean ± SD)	42.0 ± 11.4	41.0 ± 9.7	0.55^a^
Menstruation day (median, quartile range)	6 (4, 7)	11 (10, 14)	<0.001^b^
Vitreoretinal disease (*n*, %)			0.83^c^
RRD	48 (69.6)	56 (65.1)	
Choroidal RD	2 (2.9)	3 (3.5)	
Macular hole RD	7 (10.1)	10 (11.6)	
Tractional RD	4 (5.8)	4 (4.7)	
Macular hole	4 (5.8)	3 (3.5)	
Epimacular membrane	3 (4.4)	5 (5.8)	
Macular retinoschisis	1 (1.5)	5 (5.8)	
Surgeon			0.38^c^
A	3 (4.4)	9 (10.5)	
B	15 (21.7)	16 (18.6)	
C	17 (24.6)	19 (22.1)	
D	29 (42.0)	30 (34.9)	
E	5 (7.3)	12 (14.0)	
Operative bleeding (*n*, %)	10 (14.5)	9 (10.5)	0.45^c^
Postoperative bleeding (*n*)	None	None	—

SD: standard deviation; RRD: rhegmatogenous retinal detachment; RD: retinal detachment; ^a^*p* values tested by Student's *t*-test; ^b^*p* values tested by Wilcoxon; ^c^*p* values tested by chi-square.

**Table 2:** Univariate and multivariate odds ratios (OR) and 95% confidence interval (CI) for operative bleeding.

	Univariate	Model 1	Model 2
Day of menstruation	1.01 (0.93, 1.10)	1.00 (0.92, 1.09)	—
Menstruation group	0.69 (0.26, 1.81)	—	0.71 (0.27, 1.86)
Age	0.98 (0.94, 1.03)	0.99 (0.94, 1.03)	0.99 (0.94, 1.03)
Vitreoretinal disease	0.79 (0.56, 1.14)	0.80 (0.56, 1.15)	0.81 (0.56, 1.16)
Surgeon	1.07 (0.69, 1.64)	1.01 (0.65, 1.57)	1.00 (0.64, 1.56)

Model 1: adjusted day of menstruation, age, and vitreoretinal disease.

Model 2: adjusted menstruation group, age, and vitreoretinal disease.
